# Supplementary material for: Finite-time attitude tracking control for spacecraft based on backstepping method with input saturation
Source: PLoS One. 2025 Jun 18;20(6):e0326150. doi: 10.1371/journal.pone.0326150 (PMC12176304; doi:10.1371/journal.pone.0326150)
Supplement: Attachment — Submitted filename: Response to Reviewers.docx [file pone.0326150.s001.docx]

ACADEMIC EDITOR:

1)The reviewer raised various comments regarding the presentation and the technical requirements of this paper. These comments are critical, and the authors should address them thoroughly. Based on my evaluation, this paper addresses the attitude tracking control for spacecraft with input saturation. However, the input saturation control problem has been widely considered in the control literature. Relevant works include Robust consensus control of second-order uncertain multiagent systems with velocity and input constraints, controllers for multiagent systems with input amplitude and rate constraints and their application to quadrotor rendezvous. The authors should provide a comparison with these studies to highlight distinctions.

Reply 1:

This paper focuses on the finite-time attitude tracking control of spacecraft. Through the design of continuous controller based on backstepping method and combined with adaptive law to effectively suppress external interference, the hyperbolic function and auxiliary system are innovatively introduced to deal with the input torque saturation problem. While ensuring that the control torque is always lower than the preset threshold, the attitude error is converged in finite time and without jitter. Compared with the existing research: Controllers for multiagent systems with input amplitude and rate constraints and their application to quadrotor rendezvous, for the input amplitude/rate constraints of UAV systems, the algorithm is constructed by using saturation function and damping term, relying on Barbalat lemma and graph theory to achieve group coordination, but its control goal is only asymptotic stability, and the finite-time convergence characteristic is not achieved; Robust consensus control of second-order uncertain multiagent systems with velocity and input constraints focuses on the robust consensus control of second-order uncertain multiagent systems, based on fixed-time stability theory and Lyapunov function to deal with velocity and input constraints, but its controller introduces discontinuity due to $\mathrm{sgn}()$ function and piecewise saturation function, which may cause jitter problems in the actual system. Therefore, this study has significant differences in theoretical methods, application scenarios and implemented functions.

2)In addition, there are some typos scattered throughout this paper, such as (23). Below (4), "Where" should be corrected to "where".

Reply 2:

Based on the expert advice, the "Where" in formula (23) was corrected to "where" and similar questions were also modified.

Reviewer #1: In this paper, the finite-time attitude tracking control problem for spacecraft based on the backstepping method is addressed. Some comments are given to improve the paper quality.

A. There are some grammar errors in the text. For example, in page 2, the main contributions of the results in this paper are: (i) The first controller can inhibit external disturbances requiring no information about them.

Relay 1:

Based on the expert's advice, this sentence has been revised. For details, please see the third line of the fourth paragraph of the first section.

B. In Nonlinear model and problem formulation, the problem description is not clear enough.

Relay 2:

Based on the expert suggestions, the problem description has been supplemented. For details, please see the first two paragraphs of Section 2.2.

C. How to makes the attitude error e always satisfy ||e||< 1?

Relay 3:

From Rodrigues' formula, for any $Q=R_{t}^{T}R_{c}\in\text{SO(3)}$, there exists $x\in R^{3}$ with $\left\|x\right\|\leq\pi$ such that

$Q=\exp\hat{x}=I+\frac{\sin\left\|x\right\|}{\left\|x\right\|}\hat{x}+\frac{1-\cos\left\|x\right\|}{\left\|x\right\|^{2}}\hat{x}^{2}$ (1)

Substituting (1) into $e=\frac{1}{2\sqrt{1+\text{tr(}R_{e}\text{)}}}(R_{e}-R_{e}^{T})^{\vee}$, we obtain

$\left\|e_{R}\right\|^{2}=\sin^{2}\frac{\left\|x\right\|}{2}=4\sin^{2}\frac{\left\|x\right\|}{4}\cos^{2}\frac{\left\|x\right\|}{4}$ (2)

From formula (2), we can see that the attitude error $e$ always satisfies $\left\|e\right\|\leq1$.

D. The article does not provide detailed technical means for handling input saturation. From the definition of Eq.31, how to ensure that control signals do not enter saturation illegally?

Relay 4:

Formula (31) is:

(1)

where $k_{1}$, $k_{2}$, $k_{3}$ are control gains, and the hyperbolic tangent function has the following characteristics:

$\begin{matrix} -1<\tanh(x)<1 & \forall x\in R \end{matrix}$ (2)

Regardless of how the system state changes, each item of the control signal is constrained by, so that it changes within the following range:

$\left\|u\right\|\leq k_{1}+k_{2}+k_{3}$ (3)

Therefore, the control signal will not illegally enter the saturation state.

E. How to show the convergence of the system signals in the simulation? More comparison results should be given to show the advantage of this work.

Relay 5:

Based on the expert suggestions, simulation results have been added, see the section below Figure 5 in Section 4 for details.

F. Many works have been done to deal with the input saturation issues, such as event-triggered adaptive neural network tracking control for uncertain systems with unknown input saturation based on command filters, and adaptive command filtered backstepping tracking control for auvs considering model uncertainties and input saturation. So, how to demonstrate the innovation of this article.

Relay 6:

This paper proposes a finite-time attitude tracking controller based on backstepping. Through the improved adaptive law and hyperbolic function design, while ensuring the input saturation constraint, it also avoids the jitter problem caused by event-triggered switching in the article Event-triggered adaptive neural network tracking control for uncertain systems with unknown input saturation based on command filters. At the same time, the hyperbolic function and the auxiliary dynamic system are innovatively combined, and the control torque is directly constrained through rigorous mathematical derivation. There is no need for the complex event-triggered neural network in Event-triggered adaptive neural network tracking control for uncertain systems with unknown input saturation based on command filters to approach the uncertainty of input saturation and the parameter design of the command filter in Adaptive command filtered backstepping tracking control for auvs considering model uncertainties and input saturation, which significantly reduces the complexity of the controller. At the same time, compared with the above two articles, the method in this paper improves the convergence speed and ensures convergence within a finite time, and the basic convergence time is within 20s.
