# Supplementary material for: Finite-time attitude tracking control for spacecraft based on backstepping method with input saturation
Source: PLoS One. 2025 Jun 18;20(6):e0326150. doi: 10.1371/journal.pone.0326150 (PMC12176304; doi:10.1371/journal.pone.0326150)
Supplement: Attachment — Submitted filename: Response_to_Reviewers_auresp_2.docx [file pone.0326150.s002.docx]

**ACADEMIC EDITOR:**

One review has been received for the revised paper. The reviewer remains unsatisfied with the revision and raises the following concerns: (1) the manuscript still contains numerous grammatical errors, (2) the benefits of the proposed approach compared to existing work are not clearly articulated, and (3) the paper lacks reliable comparative results.

In addition, I find that the authors have not addressed my previous concerns satisfactorily. In particular, the issue of input saturation control has been extensively studied in the control literature. Relevant works include "Robust consensus control of second-order uncertain multi-agent systems with velocity and input constraints," as well as studies on controllers for multi-agent systems with input amplitude and rate constraints and their application to quadrotor rendezvous. These references should be included in the revised manuscript to provide a more balanced and fair justification of the proposed approach.

Reply (1):

According to the editor’s suggestion, the part of grammatical errors has been revised, and the specific types of corrected errors are related to tense unification (e.g., develop→developed) and correlative collocation (e.g., the \ a), as shown in the green-highlighted parts of the text.

Reply (2):

At the suggestion of the editors, a corresponding narrative has been included in the article in the yellow highlighted sections on page 1, paragraph 2, page 2, paragraph 1 and page 2, paragraph 2.

Reply (3):

At the suggestion of the editors, some references have been added to question (2) as a means of supporting the thesis statement of the paper, as detailed in the article in the yellow highlighted sections on page 1, paragraph 2, page 2, paragraph 1 and page 2, paragraph 2. And this paper combines the hypercurved tangent function with the auxiliary system to design a finite-time input saturation controller. Although the introduction of parameters makes the system response speed slightly slower than that under non-saturation conditions, showing a certain degree of conservatism, compared with the high-frequency jitter caused by the sign function (such as SGN) or piecewise saturation function used in some existing finite-time control methods, the controller in this paper maintains continuity in design and effectively avoids high-frequency switching and jitter of the control signal. More importantly, under the condition of limited physical output, the controller can still achieve high-precision attitude tracking within 20 seconds without any overshoot, showing good robustness and adaptability, ensuring good convergence performance when disturbances exist, output limitations and controller continuity coexist, and has significant advantages.

**Reviewer #1:**

Although the paper answered some of the questions, the quality of the paper did not improve significantly. Specifically manifested as
1. The paper still contains a large number of grammar errors
2. Although the control strategy used in this paper can handle input saturation, it will result in the controller being particularly conservative, especially when considering the values of k1 and k2. What are the benefits of this approach compared to existing achievements
3. The paper still does not provide reliable comparative results, and more references need to be added to explain the research purpose

Reply 1:

According to the reviewer’s suggestion, the part of grammatical errors has been revised, and the specific types of corrected errors are related to tense unification (e.g., develop→developed) and correlative collocation (e.g., the \ a), as shown in the green-highlighted parts of the text.

Reply 2:

According to the review expert's suggestion, the following explanation is made: This paper combines the hypercurved tangent function with the auxiliary system to design a finite-time input saturation controller. Although the introduction of parameters makes the system response speed slightly slower than that under non-saturation conditions, showing a certain degree of conservatism, compared with the high-frequency jitter caused by the sign function (such as SGN) or piecewise saturation function used in some existing finite-time control methods, the controller in this paper maintains continuity in design and effectively avoids high-frequency switching and jitter of the control signal. More importantly, under the condition of limited physical output, the controller can still achieve high-precision attitude tracking within 20 seconds without any overshoot, showing good robustness and adaptability, ensuring good convergence performance when disturbances exist, output limitations and controller continuity coexist, and has significant advantages.

Reply 3:

Based on the recommendations of the reviewers, the appropriate references have been added to the article, as detailed in the article in the yellow highlighted sections on page 1, paragraph 2, page 2, paragraph 1 and page 2, paragraph 2., concerning the following references:

Reference 11: Controllers for Multiagent Systems With Input Amplitude and Rate Constraints and Their Application to Quadrotor Rendezvous

Reference 19: Robust consensus control of second-order uncertain multiagent systems with velocity and input constraints
